# Efficient Control of Epidemics Spreading on Networks: Balance between Treatment and Recovery

**DOI:** 10.1371/journal.pone.0063813

**Published:** 2013-06-04

**Authors:** Katarzyna Oleś, Ewa Gudowska-Nowak, Adam Kleczkowski

**Affiliations:** 1 M. Kac Complex Systems Research Center and M. Smoluchowski Institute of Physics, Jagiellonian University, Kraków, Poland; 2 Department of Computing Science and Mathematics, University of Stirling, Stirling, United Kingdom; Universidad de Zarazoga, Spain

## Abstract

We analyse two models describing disease transmission and control on regular and small-world networks. We use simulations to find a control strategy that minimizes the total cost of an outbreak, thus balancing the costs of disease against that of the preventive treatment. The models are similar in their epidemiological part, but differ in how the removed/recovered individuals are treated. The differences in models affect choice of the strategy only for very cheap treatment and slow spreading disease. However for the combinations of parameters that are important from the epidemiological perspective (high infectiousness and expensive treatment) the models give similar results. Moreover, even where the choice of the strategy is different, the total cost spent on controlling the epidemic is very similar for both models.

## Introduction

Networks can provide a good representation of how individuals interact [1]–[3]. Despite many simplifications, models based upon network structures have successfully been used in many applications [4], [5] including spread of rumours and news [3] and computer viruses [1]. A particularly important application of network models has been in epidemiology [6]–[10] of plant, animal and human pathogens [11]–[13]. Modelling in epidemiology plays an important role: It allows us to estimate the scale of the epidemic, to predict how far the disease could spread and to design effective ways of control. All these tasks need to be achieved despite the fact that in many cases we are not able to observe the whole process and/or measure all relevant parameters [14]. The state of individuals, whether they are susceptible, infected and pre-symptomatic, infected and symptomatic or recovered, is in particular often difficult to ascertain [15]. Despite these uncertainties it is possible to use modelling to design effective control measures leading to the lowest overall cost of the epidemic outbreak [16]–[19] and a number of studies have used network models to address this issue [14], [20]–[23].

Economic and behavioural aspects influence the spread of disease and affect the choice of a control strategy. For instance, if the treatment does not cost anything, the best strategy is to control the whole population. Contrarily, for very expensive control measures it might be better to refrain from treatment at all. Optimisation of total disease costs, including palliative cost associated with disease cases and cost of appropriate control measures, leads to appearance of three basic strategies [20]: The Global Strategy (GS) whereby all individuals are treated regardless of their status can be contrasted with the Null Strategy (NS) when the public authorities completely refrain from preventive treatment and concentrate on palliative treatment of cases. The Local Strategy (LS) emerges for intermediate costs of treatment. In this case, not only detected symptomatic individuals are treated preventively, but the treatment includes also their neighbours.

The work so far has concentrated on the role of processes associated with disease spread on the broad choice of the treatment strategy [20] and on the details of the local strategy [21]. However, the spontaneous recovery also may affect the results and in the current paper we explore this dependence in detail.

We extend our results to two contrasting and yet complementary models in which we either treat individuals that have been through the disease or not. Whether the removed individuals (i.e. those who have been through the disease but then spontaneously recover or die) are part of the treatment plan depends on the type of the disease agent. The key factor in choosing the right model is whether it is possible – and desirable – to distinguish such individuals from those who are susceptible. If the removed class is identified with dead individuals, the distinction is very clear. However, if the removal means recovery and immunity, it might not be possible to identify those who are immune. For example, many people might not want to report that they have been through the infection, or the disease symptoms might be relatively mild. For animal diseases, immunological testing might be the only way to identify such individuals, but this leads to increased costs and test results might not be reliable. In other situations, we might know the status of the individual, but might not be able to target the treatment to susceptible and infected individuals. Plant and crop diseases might serve as an example here, whereby it might be easier to treat the whole field regardless of whether some plants there are already immune to the disease.

Although such individuals do not contribute to the spread of the disease, the cost of treating them affects the economic side of the evaluation and therefore leads to changes in the design of the optimal strategy. We study this case in our paper and show that although there is a difference in the choice of the strategy (LS vs. GS) and the resulting number of treated individuals, there is only a small difference in the overall total cost of the epidemic.

## Methods

**Figure 1:**
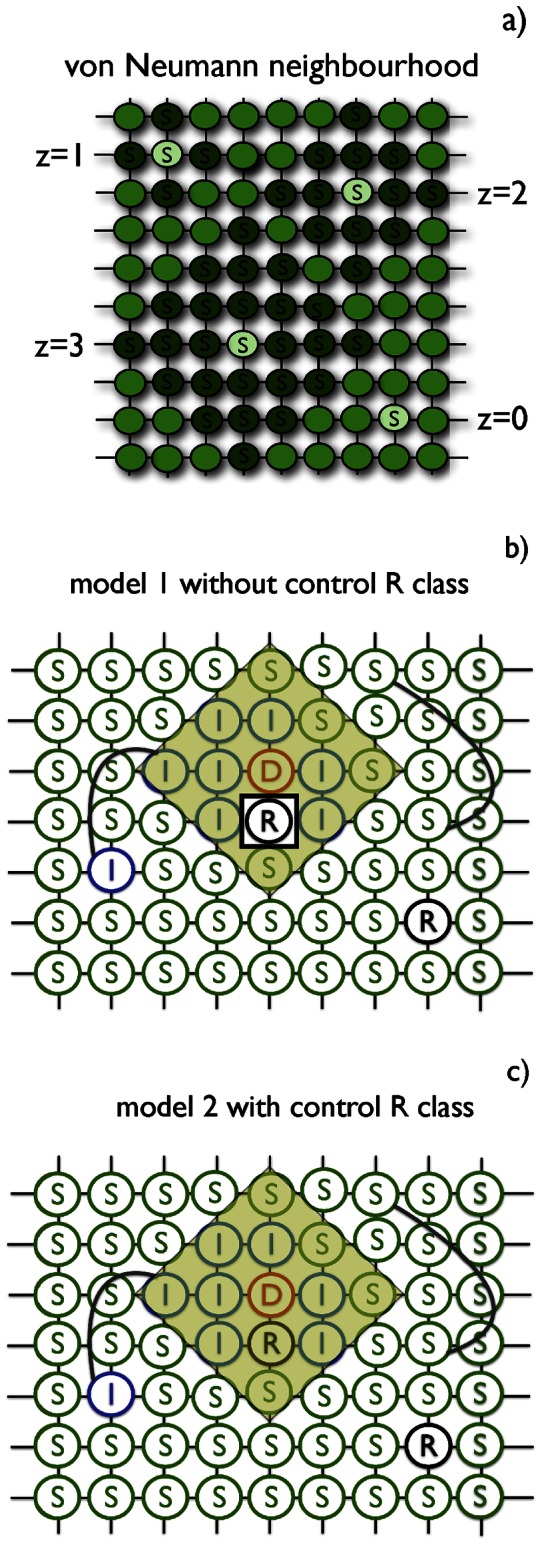
(a) Definition of the von Neumann neighborhood of different values of order 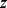, as used in the simulations and analysis. (b) Illustration of spread of a disease (model 1) on a regular network with additional randomly chosen long-range links represented by curved lines (approximation of a small-world network). The applied control of radius 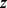 is centered on node **D** (yellow shaded area). Note that in model 1 the **R** individuals are excluded from the control and thus non-treated. (c) Representation of model 2: All individuals contained in the control neighbourhood of order 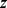 are preventively treated and moved to **V** class. In both models treatment does not take into account individuals connected by additional long-range links. S, I, D, R symbols stand for Susceptible, Pre-symptomatic, Symptomatic and Recovered, respectively. The order 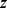 of infection neighbourhood equals 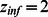 in (b) and (c).

We assume that individuals are located at nodes of a square lattice that represents geographical distribution of hosts, see fig. 1. On this lattice, we define a local infection neighbourhood of order 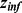 as a von Neumann neighbourhood. In that neighbourhood 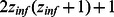 individuals are included, involving the central one. We additionally define 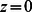 as corresponding to this central individual, which means that this individual is not in contact with anyone, while 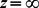 corresponds to the whole population, see fig. 1. To increase realism of our analysis, we also consider the small-world model [24], [25] which adds a certain number of links among randomly chosen nodes, thus adding some long-range connections to the regular lattice ones [24]. Although the disease can spread along these long-range links, we assume that they are so difficult to identify that they are not included in any treatment strategy (see below).

**Figure 2:**
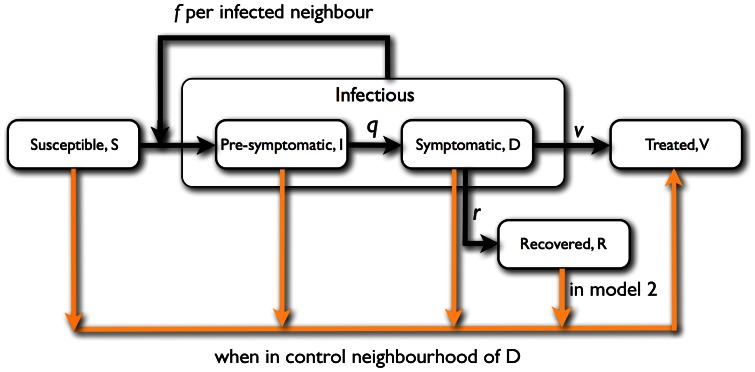
Model scheme of disease transition (black lines) and control (orange lines). In model 2 there is a possible transition between recovered (R) and treated (V) class when R-individual is in the control neighbourhood of any symptomatic D-individual.

The epidemiological SIDRV model is a standard SIR (Susceptible-Infected-Removed) model [26], modified to account for latent period and preventive and responsive treatment (fig. 2), see also [21]. Taking into consideration the latent period, the infectious class is now composed of two separate, pre-symptomatic and symptomatic classes (**S**, **I**, **D**, **R** and **V**, respectively). Number of individuals in each class is denoted by 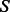, 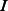, 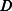, 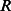, and 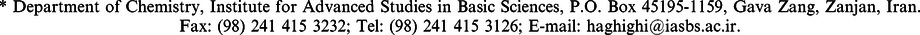, respectively, and 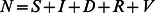 is the total constant number of individuals in the population.

Initially, all individuals are assumed to be susceptible (**S**). The epidemic is initiated by an introduction of few infected but pre-symptomatic (**I**) individuals, which are located randomly and uniformly over the whole network. Each infected individual is in contact with a fixed number of other individuals in its infection neighbourhood 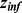. These connections do not change during the epidemic. The disease is transmitted along these contact routes with probability 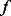 per contact. Upon a successful infection, the susceptible individual moves to the pre-symptomatic class.

Each infected pre-symptomatic individual moves to a symptomatic class (**D**) with probability 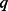. Detected individuals still can infect other individuals. Subsequently, each detected individual can spontaneously move to a removed class (**R**) with probability 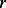. However, detection also triggers a control event with probability 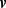 and subsequently a number of individuals selected from the von Neumann neighbourhood of order 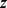 centered at the detected individual move to a treated class (**V**); for details see below. Neither **R** nor **V** can infect or be re-infected any more.

According to the responsive treatment two versions of the SIDRV model have considered: (i) model 1 with control of all individuals in selected area except removed (**R** class), see fig. 1b, and (ii) model 2 with control of all individuals in selected area regardless of their status (and thus including **R**), see fig. 1c.

The control event is localized within a von Neumann neighbourhood of order 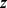 centred on a symptomatic individual. The order of control neighbourhood, 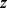, can be different than the order of the infection neighbourhood, 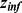, and is typically found larger. Thus, a group of individuals in the treatment neighbourhood consists of a mixture of susceptible, infected pre-symptomatic, infected symptomatic and recovered individuals (preventive treatment). We have extended the definition of control neighborhood size in order to include the situation when no control is applied, 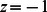.

### Simulations

All simulations have been performed on the lattice of 200 by 200 individuals with periodic boundary conditions. Simulations started with 40 initial infected foci, which corresponds to 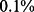 of the total population.

Control size, 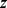, has been varied, while other parameters (such as 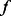, 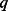, 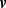, 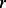, 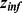,) have been kept constant. Each simulation has been run until 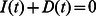, which means that no infection can occur afterwards. At the end of the run all 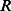 and 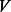 individuals have been counted, yielding information about severity of the epidemic as well as effectiveness of the treatment involved.

### Effectiveness of control strategies

The effective control strategy is found by taking into account severity of the epidemic and its financial implications. In order to quantify the effectiveness of different control strategies we introduce the severity index, 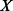 [15], [20]. By seeking the minimum values of 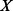, we find which strategy is optimal.

The severity index, 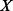, includes two terms corresponding to the cost of infection and control. First term describes costs associated with death, absence in work, lower productivity etc., whereas second term includes costs of vaccine, quarantine, transport of drugs to infection foci, etc. We assume that 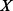 is a linear combination of number of individuals which have gone through disease and recovered (**R**) and treated individuals (**V**).

We measure 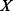 in units of a number of single infected individuals, so that:
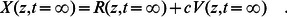
(1)


Here 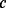 represents a cost of treatment relative to the cost of infection and 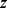 stands for the control neighbourhood size. Both 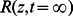 and 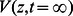 are counted at the end of a single simulation run.

Effective strategy is equivalent to the minimal value of 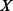, which means that the epidemic is stopped at the manageable cost. In our simulation, the minimization of the severity index has been achieved by sweeping through different values of control neighbourhood size, 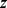 while keeping other parameters constant. Once 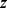 is set, we let the system evolve and then compute the value of 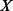 in the stationary state. We repeat this operation 100 times and then we denote with 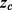 and 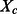 the average values, of 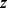 and 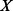, corresponding to the minimum of 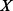, so that
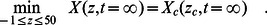
(2)


## Results

**Figure 3:**
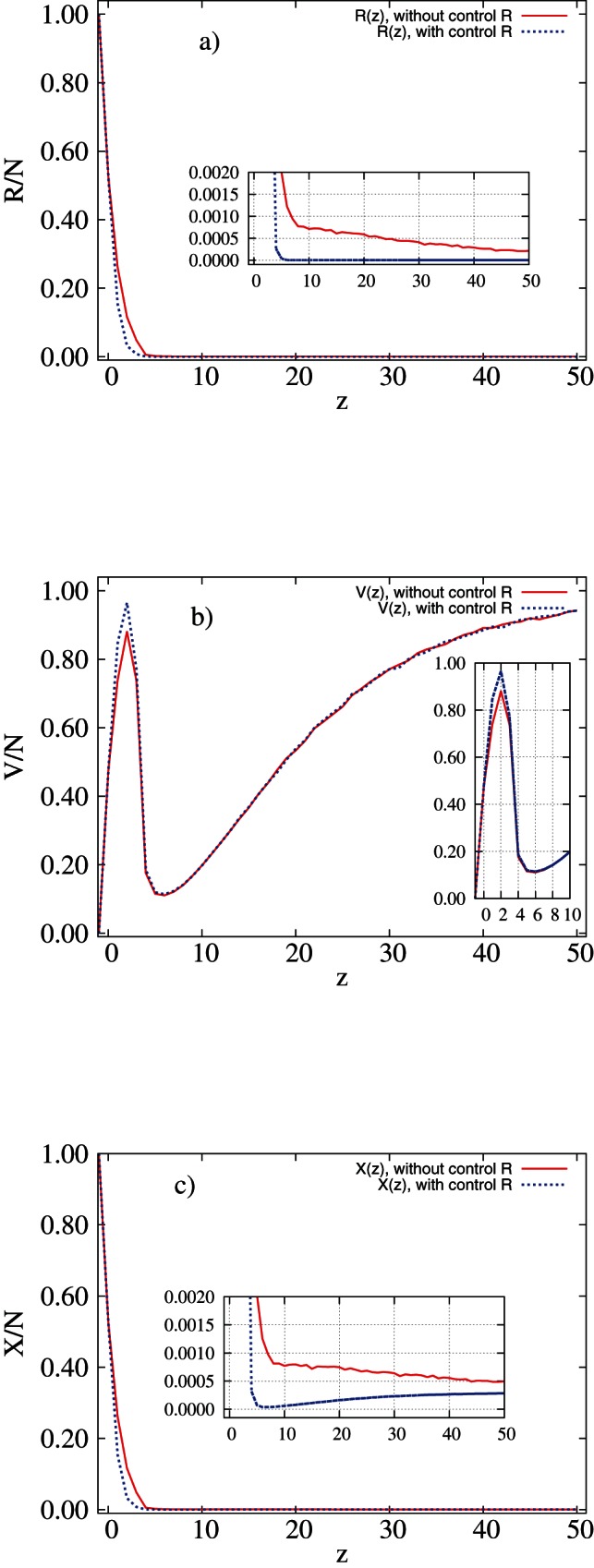
(a) The proportion of recovered individuals, 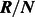, (b) the fraction of treated (controlled) individuals, 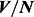 and (c) the total cost of epidemic as a fraction of the system size, 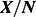, for 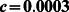 and various control sizes 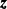. Red solid line: model 1; blue dotted line: model 2. Results of simulations with parameters 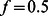, 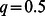, 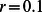, 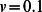 and 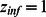 performed on regular networks. Inserts show the relevant magnifications of the graph.

In the absence of control, the disease will either progress through the population until it exhausts a large part of initially susceptible population (for large values of the infection probability 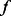) or it will quickly stop spreading (for small values of 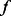). As control is applied in extended neighbourhood of radius 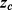 centred at a symptomatic individual, the number of recovered (**R**) individuals declines rapidly, see fig. 3a. Models 1 and 2 examined in this work differ in the way they treat or not treat the recovered class, **R**, cf fig. 1 We observe the same behaviour for both considered models (with and without treating **R** class). However, when we allow the control of **R** individuals (model 2), the proportion of recovered declines faster than in model 2, see fig. 3a (insert). The proportion of preventively treated individuals, **V**, in both models is similar for the whole range of control size, 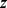. With increasing control neighbourhood, V(z) grows very quickly, then drops near 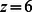 and finally rises monotonically till 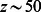 (fig. 3b). Combination of these two relationships, 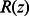 and 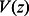, according to eq(1), gives total cost of epidemic, 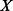, as a function of 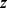, see fig. 3c. For a very low treatment cost, e.g. 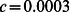, total cost of control of epidemic, 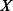, is almost equal for both models, with difference less than 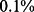, see fig. 3c (insert). The choice of optimal strategies is different for model 1 (GS) than for model 2 (LS), although the corresponding X values are similar. In model 1 the minimal value of X corresponds to the highest value of control size, 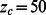 (GS), whereas in model 2, the minimum is identified with 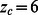, (LS) fig. 3c.

### Regular networks – influence of recovery rate, 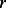 on control strategies

**Figure 4:**
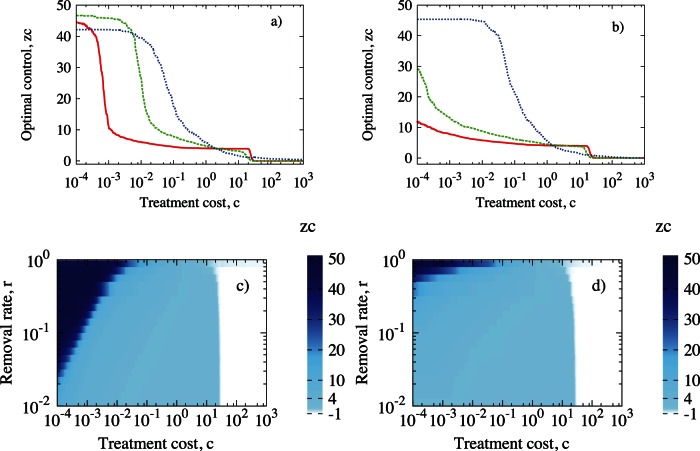
Control size 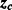 as a function of the treatment cost 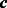 ((a) and (b)) and as a function of the recovery rate, 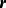, and the treatment cost, 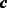 ((c) and (d)) for model 1 (left column) and model 2 (right column). In (a) and (b) 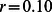 (red line), 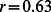 (green dashed line), 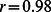 (blue dotted line). All simulations done on regular networks with parameters 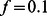, 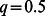, 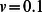, 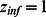.

**Figure 5:**
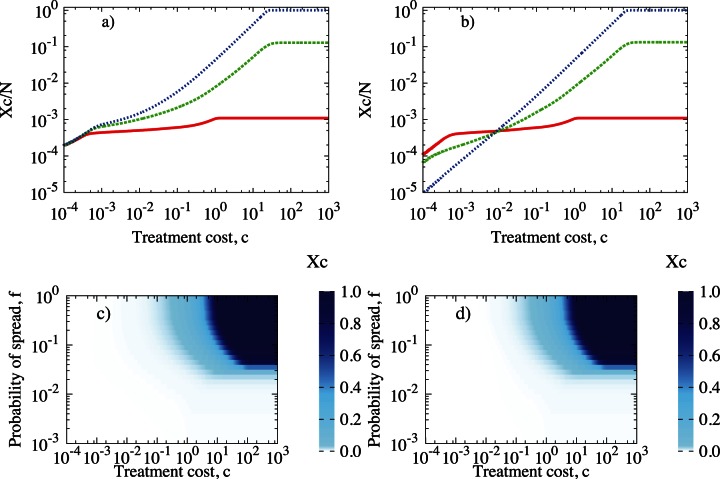
Total cost of epidemic at optimum, 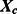, as a function of the treatment cost 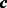 ((a) and (b)) and as a function of both infectiousnes, 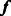, and cost, 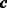 ((c) and (d)) for model 1 (left column) and model 2 (right column). In (a) and (b) 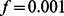 (red line), 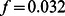 (green dashed line), 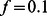 (blue dotted line). All simulations done with parameters 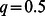, 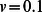, 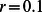, 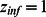. Disease spreading on regular networks.

Increasing cost of treatment, 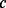, decreases the optimal control neighbourhood, 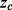. For very cheap control the optimal scenario is identified with 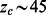 (GS) for model 1, regardless of the recovery rate, 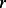 (fig. 4a). The more expensive the treatment, the higher the total costs spent on controlling outbreaks. This leads to change in optimal strategy, see fig. 4a, b. We cannot afford the preventive control of the whole population (GS) and have to shift into treating in neighbourhood of symptomatic individuals. We observe that 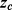 rapidly decreases with increasing costs, especially for model 1. For intermediate values of 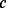, 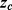 drops to 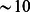 depending on recovery rate, 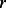. Higher recovery rate, 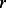, results not only in a shorter plateaux for LS (see fig. 4a, b) but also moves the plateaux towards larger control size, 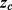. As treatment becomes more expensive, second threshold is observed that describes change from LS to NS. Although for model 2 the global strategy is selected rather than the local one as for model 1 (fig. 4b, d) for the high values of recovery rate, 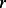 and low 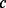, the total cost of epidemic, 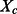, does not differ much between the two models, see fig. 5. The highest costs are associated with fast spreading diseases (large 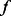) and expensive treatment (large 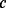) for both models (upper right part of plots in fig. 5). Slow spreading disease does not significantly affect the budget for control regardless of treatment costs (lower part of plots in fig. 5) and model selected. For model 2 the global strategy is predominantly selected for high values of recovery rate 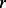 and at low 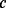, in contrast to model 1 (fig. 4b, d) where the local strategy prevails. Despite these differences, the total cost of epidemic, 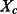, does not differ between the two models, see fig. 5.

### Regular networks – control strategies

**Figure 6:**
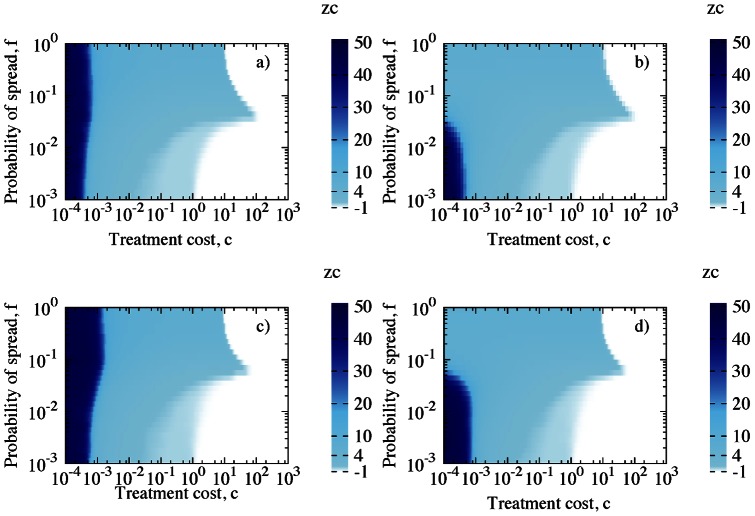
Control size, 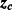, as a function of both infectiousness, 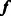, and treatment cost, 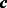, for model 1 (left column) and model 2 (right column). Simulation parameters for top panel ((a) and (b)): 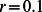; for bottom panel ((c) and (d)): 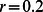; other parameters: 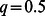, 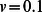, 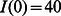, 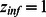. Disease spreading on regular networks.

Control size, 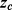 depends strongly on the cost of treatment, 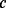, and on the infectiousness of the disease, 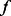 (fig. 6). For small 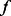 and 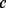, both models suggest preventive control extended to the whole population (GS) (lower left part of each plot in fig. 6). In case of highly infectious disease and low treatment costs, model 1 predicts higher effectiveness of GS whereas model 2 selects LS as an optimal solution, upper left part of each plot in fig. 6. However, in both examined models the total cost of epidemic, X, is approximately the same, see fig. 3. As treatment cost, 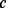, increases, LS becomes the most cost-effective strategy. LS changes to NS when 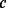 is of order 1 for small 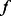 and of order 10 for high 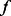, regardless of the choice of the model or the exact value of 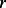, compare fig. 6a, b with fig. 6c, d.

The main difference in selection of the optimal strategy occurs for small 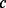. Changes in 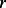 affect only low 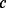 regions. Increasing 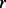 from 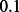 to 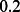 extends the region of validity of GS and moves it towards marginally larger values of 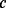 and high values of 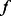, fig. 6c, d. This trend is continued for larger values of 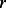, see fig. 4, and can be associated with faster removal of individuals without triggering control events.

### Small world networks – control strategies

**Figure 7:**
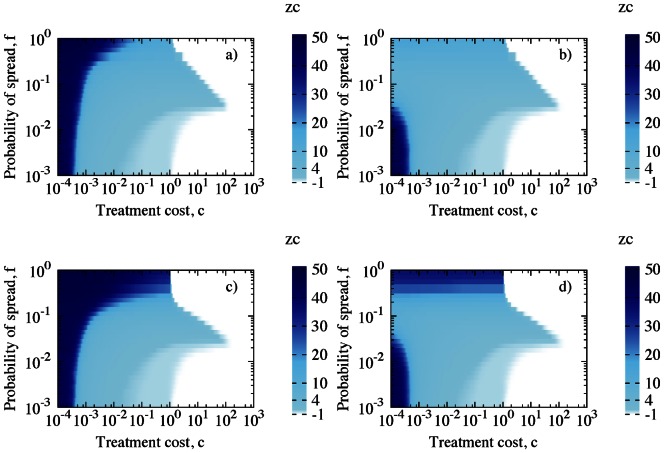
Control size, 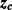 as a function of both infectiousness, 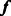, and treatment cost, 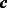, for model 1 (left column) and model 2 (right column). Number of additional links with respect to all the ones that can be added, on the top panel ((a) and (b)): 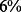, on the bottom panel ((c) and (d)) –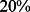. Other simulation parameters: 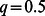, 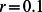, 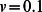, 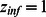. Disease spreading on small-world networks.

Addition of small-world links does not change the behaviour for small 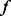 and 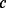. However, there are substantial differences for large 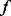 and the effect differs for the two models. Introducing disorder into the topology by adding long-range links changes ranges of optimal strategy for both considered models, compare fig. 6a, b with fig. 7. In model 1 small number of links, e.g.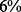, fig. 7a, extends GS when disease spreads fast and costs are higher. The small number of links 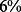 in model 2 does not change choice of control strategy, compare fig. 6b with fig. 7b, as in model 1 (top panel in fig. 7). Nonetheless, the total cost of epidemic remains almost the same. For large values of 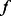, destroying spatial structure by adding 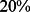 links results in only two effective strategies for highly infectious disease, GS for 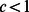 and NS otherwise, fig. 7c. The higher disorder (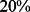 of long range links) in model 2, introduces GS when probability of spreading the epidemic, 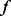, increases, fig. 7d.

## Discussion

The goal in designing cost-effective control strategy is to stop the epidemic outbreak very quickly at a minimal possible cost. In order to achieve this by using the local strategy (LS) we need to catch in the preventive control neighbourhood as many infected but pre-symptomatic individuals and to form a fire-break by treating around the infection focus. The extend of control is a crucial factor; however, it is not obvious by how much we need to enlarge the neighborhood in which preventive treatment is applied. We need to balance epidemiological and economic aspects of disease spread and control [27]. When we extend prevention to the whole population we might be able to successfully protect population from epidemic outbreaks but we will need to spend a lot of resources. On the other hand, when we apply control to too small neighbourhood, we will spend a lot but the disease will still invade the whole population. Under some conditions an optimal solution emerges in between these two extremes and can be associated with the Local Strategy; in other cases the extreme solutions (Global Strategy and Null Strategy) are optimal. As we have already shown [20], [21], the effective control neighbourhood can be chosen based on combined epidemiological and economic analysis.

The previous analyses [20], [21] left three key questions unanswered. Firstly, should we treat individuals that are already immune? Although the answer clearly depends on the nature of the disease and the treatment, some general principles can be established. This depends on the relative – economic, social and medical – cost of the preventive treatment compared to the palliative care (when we just let the disease to run its natural course). Secondly, are our results stable with respect to structural changes of the model? We illustrate the stability by considering two versions of the same model, with and without treating recovered **R** individuals. Finally, it is the dependence of the results on the actual recovery rate, 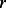. In real-life applications it is difficult to distinguish between individuals that have been through the disease and those who do not. It is therefore very important to check whether the model and the resulting policy implications are robust with respect to the potential uncertainties. We show that this is the case in general but also identify the region of the parameters when the two models have different behavior (small 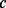, large 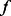).

Two contrasting cases can be distinguished in answer to the first question. If the treatment is costly and/or may lead to complications, the authorities might want to invest in testing individuals in order to find out who is and who is not naturally immune. This would identify individuals in the **R** class who then might not be offered the treatment. Contrariwise, if it is not immediately obvious what the actual status of the individual is and testing is expensive, lengthy or unreliable, the authorities might decide to treat all individuals regardless of their status. Our results from this paper suggest that the choice of the strategy depends on whether treatment includes or excludes **R** but the total budget spent on controlling epidemic remains similar for both models.

Secondly, in the most important region of parameter space, corresponding to expensive preventive treatment and a highly infectious disease, both models yield very similar scenarios (right part of fig. 4c, d). Thus, the results appear to be stable with respect to structural changes of the model. Where the difference is marked, for low 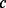 and high 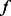, the models suggest a different choice of strategy (GS for model 1 and LS for model 2). However, we also found that in this case the economic outcome of either GS or LS is very similar (see fig. 3c).

Thirdly, the main effect of increasing 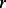 is to shift the boundary between the GS and LS for small 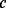, rendering the GS less attractive as 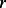 decreases – and the infectious period increases. For model 2 (without treatment of **R**) the area of preference of GS over LS is limited to very small values of 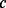. Thus, the longer the infectious period, the more likely the local strategy is to work. The boundary between LS and NS for large values of 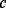 remains unchanged.

Addition of long-range links enlarges the region of applicability of GS towards higher 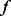 and 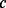 for both models. The large number of randomly placed long-range links destroys spatial structure of spreading the pathogen and causes that it spreads mostly globally so that LS is no longer effective option of control the epidemic.

The results obtained in this paper can be used for those diseases for which spread is dominated by local transmission or by a mixture of local and long-range links. Examples include human (notably SARS [28] and influenza [29]–[31]), animal (foot-and-mouth disease [32]) and plant diseases (citrus canker [33], sudden oak death [34]–[36] and rhizomania of sugar beet [37], [38]). Although our model assumes a simple network structure, we believe that the results can be generalised to more complex, but also more realistic networks, including social networks [31]. This work can also be extended in several ways. The most interesting will be the SIRS model, in which after some period of immunity to the disease individuals become susceptible again and could catch a disease few times; with influenza [29]–[31] and sexually-transmitted diseases [39], [40] being the best examples.
